# One-Step Transepithelial Topography-Guided Ablation in the Treatment of Myopic Astigmatism

**DOI:** 10.1371/journal.pone.0066618

**Published:** 2013-06-17

**Authors:** Aleksandar Stojanovic, Shihao Chen, Xiangjun Chen, Filip Stojanovic, Jia Zhang, Ting Zhang, Tor Paaske Utheim

**Affiliations:** 1 SynsLaser Kirurgi, Oslo and Tromsø, Norway; 2 Eye Department, University Hospital North Norway, Tromsø, Norway; 3 School of Optometry and Ophthalmology and Eye Hospital, Wenzhou Medical College, Wenzhou, Zhejiang, China; 4 Key Laboratory of Vision Science, Ministry of Health P.R. China, Wenzhou, Zhejiang, China; 5 Faculty of Medicine, University of Tromsø, Tromsø, Norway; 6 Department of Medical Biochemistry, Oslo University Hospital, Oslo, Norway; Medical University Graz, Austria

## Abstract

**Purpose:**

To evaluate one-step topography-guided transepithelial ablation in the treatment of low to moderate myopic astigmatism using a 1KHz excimer laser.

**Methods:**

Retrospective study of 117 consecutive eyes available for evaluation 12 months after surgery. Pre- and post-operative visual and refractive data as well as post-operative pain and haze were analyzed. A novel technique integrating custom refractive- and epithelial- ablation in a single uninterrupted procedure was used.

**Results:**

The mean pre-operative spherical equivalent (SE) and the mean cylinder were: –3.22 diopters (D) ±1.54 (SD) (range –0.63 to –7.25 D) and –0.77 D ±0.65 (range 0 to –4.50 D), respectively. At 12 months after surgery: no eyes lost ≥2 lines of corrected distant visual acuity (CDVA). Safety and efficacy indexes were 1.27 and 1.09, respectively. Uncorrected distant visual acuity (UDVA) was ≥20/20 in 96.6% of the eyes. Manifest refraction spherical equivalent was within ±0.5 D of the desired refraction in 93.2% of the eyes. Average root mean square (RMS) wavefront error measured at central 6 mm, increased from 0.38 pre-operatively to 0.47 µm post-operatively. Refractive stability was achieved and sustained 1 month after surgery. No visually significant haze was registered during the observation period. Post-operative pain was reported in 4.5% of patients.

**Conclusions:**

One-step transepithelial topography-guided treatment for low to moderate myopia and astigmatism performed with a 1 KHz laser, provided safe, effective, predictable and stable results with low pain and no visually significant haze.

## Introduction

Due to its lower impact on corneal biomechanical stability and lower risk of dry eye[1], [2] compared to LASIK, surface ablation is often used in cases with thin corneas, recurrent erosion, predisposition for trauma, or in patients who are anatomically or psychologically unsuitable for use of microkeratome.[3] Post-operative haze,[4]–[6] slow visual recovery[7], [8] and post-operative discomfort,[9], [10] all inherent to the traditional photorefractive keratectomy, are now better controlled with modern surface ablation protocols. Smooth ablation achieved by small-Gaussian-beam, high-frequency lasers, pre-operative use of mitomycin-C, post-operative protection from UV-radiation and dietary supplementation with vitamin-C have previously reduced the incidence and severity of post-operative haze after surface ablation.[11]–[14]_ENREF_10 Refinement of de-epithelialization techniques and pre- and post-operative medication continues to decrease the severity of post-operative discomfort[15] and to increase the speed of visual recovery.[16], [17] The current transepithelial topography-guided ablation technique has been reported earlier for treatment of irregular astigmatism.[18]–[20] To our knowledge, not only has the combination of one-step transepithelial ablation, topography-guided custom ablation and 1000 Hz-laser technology never been published before, but there are few reports concerning any of the mentioned technologies; transepithelial ablation,[21]–[23] topography-guided ablation,[24]–[30] or use of 1000 Hz- laser[31], [32] in routine treatments of low to moderate myopia in virgin eyes.

### Patients and Methods

The present retrospective study comprises 117 consecutive eyes of 61 patients (30 female, 31 male) who were available for evaluation at ≥12 months after treatment for myopic astigmatism, at SynsLaser Clinic in Tromsø, Norway. The mean age was 32.71±9.7 years (range 18 to 61). Patients provided written, informed consent and the study was approved by the regional ethics committee “Regional komité for medisinsk og helsefaglig forskningsetikk, Nord-Norge (REK Nord)”- P REK NORD 55/2009 Sikkerhet og effekt av refraktiv kirurgi med iVIS lasersystem: en retrospektiv analyse av kliniske data. Inclusion criteria for treatment were: age ≥18 years; no soft contact lens wear for 1 week (hard contact lens for 4 weeks) before the baseline examination; SE between –0.5 and –10.00 diopters (D) with ≤6.00 D of refractive astigmatism; stable refractive error (change of SE ≤0.50 D) for ≥2 years; and CDVA of 20/25 or better. Exclusion criteria were: eye pathology, including keratoconus or keratoconus suspect (detected by corneal topo-/tomography); previous eye surgery; glaucoma; diabetes; and systemic diseases that could affect corneal wound healing (e.g. collagen vascular diseases). Pre-operative examination included slit lamp biomicroscopy, Scheimpflug-based corneal topo-/tomography (Precisio, iVIS Technology, Taranto, Italy), visual acuity/subjective refraction (Nidek RT 2100 system, Nidek Co. Ltd., Aichi, Japan), Placido-based corneal topography and wavefront aberrometry (Nidek OPD II, Nidek Co. Ltd., Aichi, Japan), dynamic pupilometry (pMetrics, iVIS Technology, Taranto, Italy), Goldmann applanation tonometry and central ultrasound pachymetry (Corneo-Gage Plus, Sonogage Inc., Cleveland, Ohio).

The refraction, corneal anterior elevation and pachymetry maps of patients, as well as their dynamic pupilometry data were imported to “Corneal Interactive Programmed Topographic Ablation” (CIPTA, iVIS Technology, Taranto, Italy) software to generate a custom ablation plan within a treatment zone suggested by the dynamic pupilometry. The pupilometer measures pupil size and the speed of the pupillary reactions under various lighting conditions adjusted for the patient’s “life-style”. The result of the examination produces so-called “ideal entrance pupil” size, to which the distance of pupil decentration with respect to the visual axis intercept is added, resulting in the default optical zone that can be overwritten by the user. The CIPTA software uses subjective refraction and corneal topography as the basis for treatment of lower- and higher-order aberrations (HOAs), respectively (the latter limited to HOAs originating from the corneal surface). Since the treatments are calculated on the basis of topography, the manifest cylinder is superimposed on the existing toric surface. Hence the aimed regular aconic surface is a resolution of the vectors of the manifest and the corneal astigmatism. In the current study, the calculated post-operative minimal corneal residual stromal thickness was set to ≥350 µm, assuming the epithelial thickness of 65 µm. All treatments were aimed toward achieving emmetropia and within the ablation pattern the optic of the ablated lenticel were centered on the corneal intercept of the visual axis.

Ascorbic acid (C-vitamin, Weifa, Norway) and EPA/DHA (Omega-3, Nycomed, Norway) were both given 1000 mg daily 2 weeks before and 2 weeks after the surgery. Prednisolone (Prednisolone, Nycomed, Asker, Norway) 40 mg tablet and Alprazolam (Xanor, Pfizer Inc., New York, USA) 0.5 mg tablet were given orally as a single dose 30 minutes before the surgery. Diclophenac 0.1% (Voltaren, Novartis, Basel, Switzerland) and ciprofloxacine 0.5% (Cilox, Alcon, Fort Worth, Texas) eye drops were administered 15, 10 and 5 minutes before the surgery. Proparacaine 0.5% (Alcaine, Alcon, Fort Worth, Texas), 2–3 drops, were applied before, and 1–2 additional drops after the insertion of the eye speculum. Before the laser ablation, 10–15 drops of Balanced Salt Solution (BSS, Alcon, Fort Worth, Texas) chilled to the freezing point, were applied, after which the corneal surface was gently dried with a lint-free pre-shrunk Merocel until a reflective homogeneous, dry epithelial surface was achieved. After the cyclotorsional (iris and scleral vessels) and the pupil eye-tracker-registration, the treatment was performed with a 0.6-mm dual-flying-spot 1 KHz (2×500 Hz) excimer laser (iRES, iVIS Technology, Taranto, Italy) using the one-step custom transepithelial “no-touch” (cTEN, iVIS Technology, Taranto, Italy) ablation technique.

**Figure 1:**
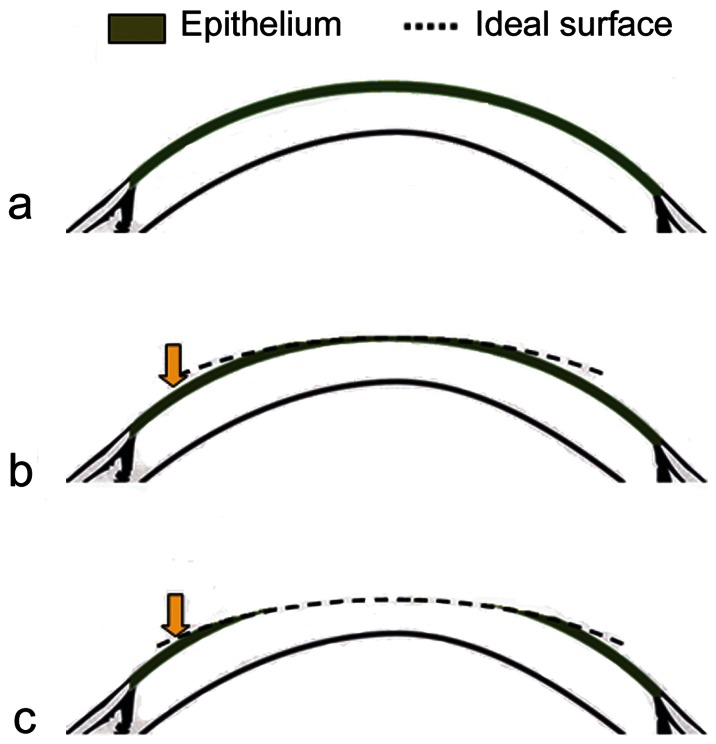
Pre-operative corneal surface with the epithelium (green) (a), is reshaped into a regular aspheric surface of desired curvature (b) and the new surface is transferred below the epithelium (c) in a single non-interrupted ablation.

The cTEN-ablation consists of a *refractive part*, which reshapes the corneal surface within the treatment zone into an aspheric regular shape of desired curvature, and a *lamellar part* of 65 µm, based on Reinsteińs measurement which showed thickest epithelium of 58.8 ±4.9 µm.[33] (Figure 1). These two parts are summed and executed in a single uninterrupted ablation, lasting typically 20–35s. With this approach, the de-epithelialized area corresponds directly to the area of the custom ablation, so only an absolutely necessary amount of epithelium is removed. The mean targeted optical zone diameter was 6.30±0.28 mm (range 6.0 to 7.6 mm) and the mean total ablation diameter was 7.72±0.62 mm (range 7.0 to 9.9 mm).

At the end 1–2 non-preserved Chloramphenicol (Novartis, Basel, Switzerland) eye drops were applied, followed by a bandage contact lens (Acuvue Oaysis, Johnson & Johnson, USA). Dexamethasone with Chloramphenicol mixture (Spersadex med Kloramfenikol, Novartis, Basel, Switzerland) eye drops QID were used the first 2 weeks, and then replaced by a low potency steroid Rimexolone 1% (Vexol, Alcon, Fort Worth, Texas) eye drops in tapering doses for another 2–4 weeks. Refrigerated 0.2% hyaluronic acid (Oxyal, SantenPharma, Solna, Sweden) was used for lubrication and lavage purposes every 10 minutes the rest of the day after the surgery and later as needed. Additionally, the patients were supplied with “comfort drops”, a single container with 0.5 ml, 1% Tetracaine hydroxide (Tetrakain minims, Chauvin, Kingston, England) in case of pain. All patients were questioned regarding postoperative pain and the need for use of “comfort drops”. _ENREF_22.

Bandage contact lens was removed from the cornea of patients between post-operative days 3 to 7 and patients were observed at 1, 3, 6, and 12 months after surgery. Post-operative examinations were similar to pre-operative examinations. Haze was defined as: grade 0.5 - trace easily seen using a slit-lamp microscope; grade 1 – trace that does not affect vision; grade 2 - dense patches affecting vision; grade 3 - dense partially obscuring iris details; grade 4 - dense haze completely obscuring iris details.

Statistical analysis was performed using SPSS 13.0. The initial Snellen visual acuity was recorded using logMAR steps. Calculation of mean visual acuity was achieved via conversion from Snellen to logMAR and back to Snellen. The outcomes were reported according to the Standardized graphs and terms for refractive surgery results.[34].

## Results

### Accountability

Among the 165 eyes of 86 patients treated for primary myopia from September 21 to December 19, 2009, 117 eyes of 61 patients (71%) were available for evaluation ≥12 months after surgery. Among those, 104 (89%), 110 (94%), and 66 (56%) eyes were also available for evaluation at 1, 3 and 6 months post-operatively, respectively.

### Baseline data

The mean pre-operative UDVA and CDVA were 20/160 (range 20/1000 to 20/25) and 20/18.2 (range 20/20 to 20/13.3), respectively. The mean pre-operative SE was –3.22±1.54 D (range –0.63 to –7.25 D) and the mean cylinder was –0.77±0.65 D (range 0 to –4.50 D).

### Efficacy

**Figure 2:**
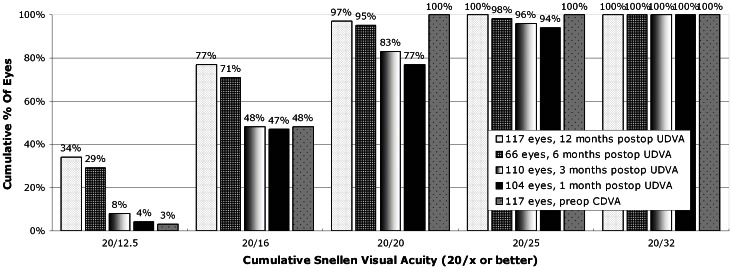
Post-operative uncorrected visual acuity vs. pre-operative best spectacle-corrected visual acuity.

Cumulative UDVA at 6 and 12 months after surgery compared to pre-operative CDVA are shown on Figure 2. Efficacy index was 0.91, 1.00, 1.09 and 1.09, at 1, 3, 6 and 12 months post-operatively, respectively.

### Predictability

**Figure 3:**
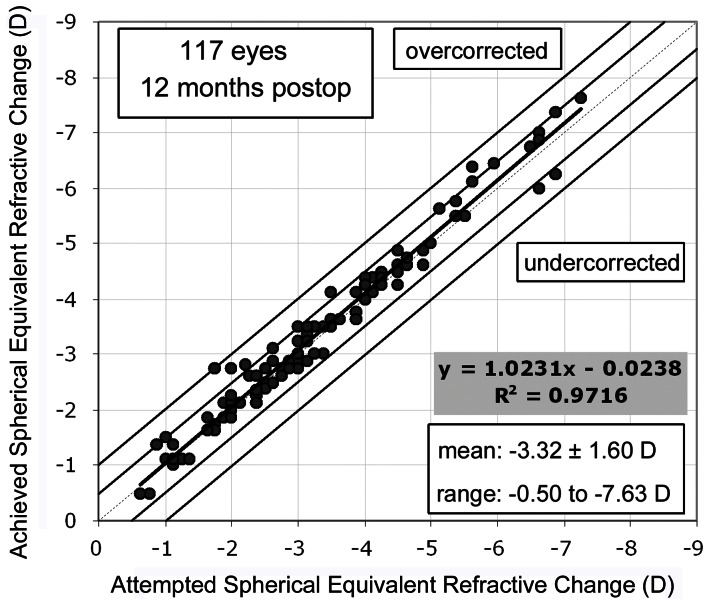
Attempted vs. achieved spherical equivalent refractive change at 12 months postoperatively.

The attempted versus achieved SE at 6 and 12 months post-operatively are shown in Figure 3. All eyes were within ±1.0 D of emmetropia, 94% of eyes were within ±0.5 D of emmetropia at 12 months and 97% of eyes had refractive astigmatism less or equal to 0.5 D at 12 months post-operatively.

### Safety

**Figure 4:**
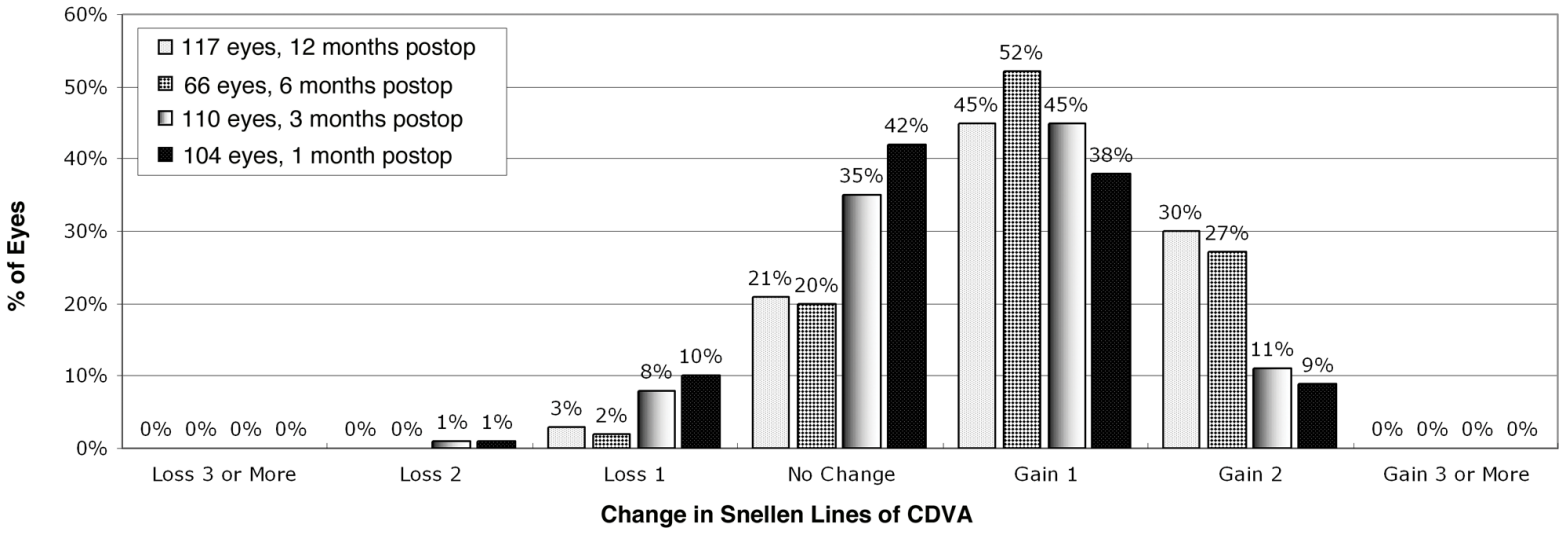
Change in lines of best spectacle-corrected visual acuity at 6 and 12 months after surgery.

Loss and gain of lines of CDVA are shown in Figure 4. The safety index was 1.27 for both 6 and 12 months.

### Stability of refraction

**Figure 5:**
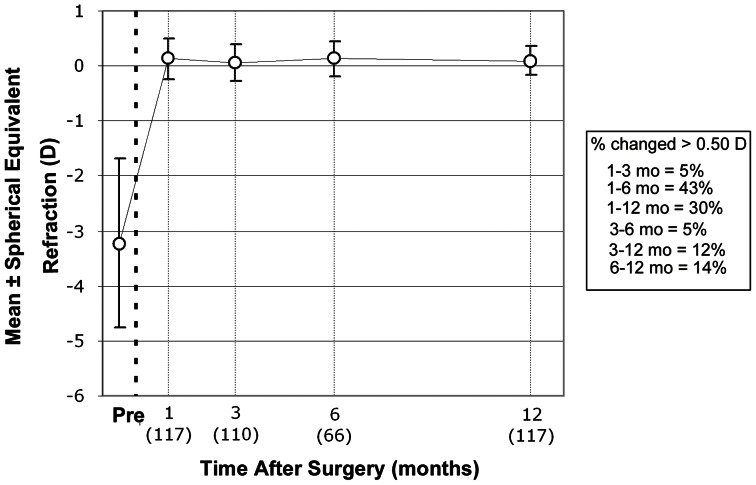
Stability of spherical equivalent. **Error bars indicate standard deviation.**

The stability of refraction is shown on Figure 5. Post-operative spherical equivalent refraction stability was reached at 1 month, with no statistically significant difference between each two follow-up points thereafter. Seven (10.6%) out of 66 eyes available for a 6 month follow-up control, regressed more than -0.50 D between 6 and 12 months post-operatively.

### Higher order aberrations

Pre-operative and 12-month post-operative average RMS of total HOAs, coma-type- (S3+5+7) and spherical- (S4+6+8) aberrations are shown in table 1. RMS of total HOAs and coma-type aberrations increased, while the spherical aberration showed no statistically significant change.

**Table 1:** Mean wavefront aberrations 12 months after surgery and pre-operatively (6 mm zone analysis, 96 eyes).

	12 months post-op	Pre-op	P value
RMS HOA (SD)	0. 4655 (0.1877)	0. 3831 (0.1801)	0.000
S 3+5+7 (SD)	0.4012 (0.1924)	0.3238 (0.1639)	0.000
S 4+6+8 (SD)	0.2005 (0.1059)	0.1761 (0.1111)	0.055

RMS: Root Mean Square; HOA: Higher Order Aberration; SD: Standard Deviation; S3+5+7: Odd Order Aberrations; S4+6+8: Even Order Aberrations.

### Pain

On post-operative day one, 4.5% of patients reported pain, as assessed by need for use of “comfort eye drops”. No complaints of post-operative pain were registered thereafter.

### Complications

No sight-threatening complications (decentration of ablation, infection, persistent epithelial defect, recurrent erosion, scaring or keratectasia) were observed. Thirteen eyes (11.1%) developed trace haze (grade ≤0.5) within 6 months after surgery, but corneas regained their pre-operative transparency without treatment within the next 6 months. No visual symptoms or loss of acuity that could be attributable to haze were found. No consistency with respect to the amount of treatment or any other preoperative parameters was found in the cases where the haze was identified.

## Discussion

The appealing idea of photorefractive keratectomy (PRK) combined with excimer laser epithelial removal instead of mechanical or alcohol debridement has been explored during the last 15–20 years in various forms, with different lasers.[35]–[38] Lamellar (non-refractive) excimer laser ablation in the form of phototherapeutic keratectomy (PTK), has been used for laser epithelial removal preceding PRK. Using this approach, some studies have demonstrated minimal keratocyte apoptosis[39], [40] and less haze,[41] as well as better visual outcomes[23] compared to mechanical epithelial debridement. Other studies, however, either did not achieve better outcomes compared to mechanical techniques,[42]–[45] or less pain compared to ethanol-assisted epithelial debridement.[46] Hence, transepithelial surface ablation has previously been limited to the treatment of highly irregular corneas.[18], [22], [47], [48] To ensure the necessary predictability of outcome that is required for routine use of the transepithelial technique in treatment of refractive errors in virgin eyes, the following advancements in excimer laser technology were necessary: 1) sufficient ablation speed to avoid corneal hydration issues; 2) even radial thickness of each ablation layer for refractive-neutral epithelial ablation, and 3) a smooth ablation surface regardless of the high total ablation depth. With the iRES 1 KHz laser, a full thickness epithelial removal can be performed in only 16 s, whilst a myopic ablation for 6 D (6.5 mm optical zone and 7.5 mm total ablation zone) requires 15 s. In conventional flying-spot lasers there is typically a linear increase in the number of pulses per mm^2^ per s (local frequency), as the ablation area decreases (towards the end of the treatment). This leads to an increased thermal effect and plume production, culminating in a lower ablation effect, which is traditionally compensated for by empirical nomogram adjustments. Meanwhile, the laser used in the current study keeps a constant local frequency of 4 Hz, i.e. the laser beam will hit the same spot of the treated area 4 times per s. Hence, the entire ablation retains a consistent local delivery of energy across the ablation area, producing a constant and even thermal effect. This is crucial for achievement of the constant thickness of each ablation layer as well as a smooth ablation surface after the ablations of high volume of tissue (when both the epithelium and the stroma are ablated within one uninterrupted treatment). Linking refractive ablation with epithelial removal by performing the corneal reshaping on the epithelium and then translating the new shape to the stroma (Figure 1), assumes compatibility of ablation rates between the two corneal layers. Published data for one laser platform show a relatively small difference in the ablation rate between epithelium and stroma (0.55 +/– 0.1 vs. 0.68 +/– 0.15 µm per pulse),[49] but the difference in ablation rate between epithelium and stroma may differ among the lasers depending on their energy fluence, shot pattern and frequency. The laser used in the present study optimizes these three parameters to minimize differences in ablation rates between the epithelium and the stroma. A former study by our research group demonstrated that transepithelial surface ablation using the iRES laser speeded reepithelialization and reduced post- operative pain compared to traditional PRK with Allegretto 400 Hz laser using Amois brush for deepithelialization.[50].

The Scheimpflug-based Precisio topographer used in the current system provides true primary elevation information acquired by triangulation. Elevation data exported from Precisio are referenced to the center of the pupil. It is well known, however, that the pupil centroid will shift with different pupil size, resulting in a registration error that may significantly affect the quality of the outcome.[51] The current system addresses this issue by employing an intra-operative laser illumination adjustment, which automatically modulates the intensity of light until the same pupil size, as registered during the Precisio data acquisition, is achieved. The “constant pupil size” during the ablation contributes to a robust registration along with the systems iris/scleral vessel dynamic cyclotorsional tracking as well as its synchronized x, y-pupil-tracking.

Using topography-guided custom ablation in treatment of virgin eyes[52] implies correction of HOAs originating only from the corneal surface. This seems to be a reasonable approach as the corneal surface is responsible for the majority of light refraction in the eye. Furthermore the corneal HOAs are static and hence more appropriate as a target for treatment than the dynamic HOAs of crystalline lens. In addition to correcting the corneal surface HOAs, the current topography-guided ablation creates a customized transition that keeps a constant dioptric gradient towards the untreated cornea, instead of employing commonly used fixed transition zone diameter. This may lead to lower regression by preventing any counterproductive epithelial remodeling.

Peer-reviewed publications from the recent five years reporting the outcomes of PRK in treatment of myopic astigmatism in virgin eyes are listed in table 2. The table shows that the safety, efficacy and predictability in our study are comparable with the best outcomes of the other studies, independent on the mode of epithelial removal or the excimer laser used. Moreover, the table shows only trace haze (grade ≤0.5). This also compares very favorably with the other studies. There is only one publication[21] reporting the outcomes using a similar approach to the current one, with the epithelial removal integrated within a single ablation. However, the treatments from that report were non-customized and only the initial 3-month results in 50 eyes were analyzed.

**Table 2:** Summarized outcomes of recent studies reporting PRK-outcomes in treatment of myopic astigmatism in virgin eyes.

Study no.		Technique	Published (y)	Follow-up (m)	Eyes(n)		SI	EI	Predictability			MMC	Haze	Epi. removal
									±0.5D	±1.0D				
1[54]		WF-guided PRK	2011	12	34		1.15	1.02	91%		97%	/	no	Amoils brush
2[55]		WF-optimize PRK	2011	12	303		1.05	1.05	99%		/	abl. dep.≥80 µm	grade ≤1	20% ethanol
3[56]		Custom PRK	2012	12	40		1.23	1.15	89%		94%	/	not significant	/
		Standard PRK			40		1.19	1.06	86%		97%	/	not significant	/
4[57]		Standard PRK	2012	12	298		1.29	0.67	83%		100%	/	not significant	Amoils brush
	Transepithelial PRK													
5[23]		Standard T-PRK	2007	8.9	37	low to moderate myopia	/	0.93	95%		/	/	not significant	PTK
					22	high myopia	/	0.84	95%		/	/	not significant	PTK
6[22]		Standard T-PRK	2009	3	15		1.17	/	/		/	/	grade <2	PTK
7[21]		T-PRK	2011	3	50		1.00	0.98	/		/	/	10% grade 1	Integrated PTK
8		Topo-guided T-PRK		12	117		1.27	1.09	94%		100%	abl. dep.≥100 µm	11% grade 0.5	Integrated PTK

WF: Wavefront; T-PRK: Transepithelial PRK; SI: Safety Index =  preoperative CDVA/postoperative CDVA; EI: Efficacy Index =  postoperative UDVA/preoperative CDVA; MMC: Mitomycin C.

One shortcoming of the transepithelial technique used in this study is the epithelial thickness estimation (estimated to 65 µm by default), instead of using the real measurement. This estimation may lead to too deep ablation and waste of corneal tissue if the real epithelial thickness is lower, or to shallower ablation and a smaller optical/treatment zone than intended if the real epithelial thickness is higher. A high-resolution corneal imaging technology, that could be expected in the near future, is likely to provide the necessary precision for measurement of epithelial thickness. This may provide an epithelial thickness map usable for custom ablation planning. In that event, an ablation plan consisting of an epithelial and a stromal component would allow for accurate compensation for any difference in laser ablation rates between these two corneal tissues, further increasing the precision of the procedure. This would address the issues of slightly irregular ablation in eyes with non-uniform epithelial thickness,[33] as described in our previous work.[53] Precise epithelial thickness mapping would also be of great value in studying the influence of different reepithelialization profiles on the refractive outcomes and would supposedly be of great help in refining the ablation design.

The idea of one-step “no-touch” laser treatment appeals to the patients because it is much quicker and more comfortable than traditional excimer laser surgery. The main appeal of this procedure to the surgeon is its perceived lack of serious complications as well as ease and speed of performance.

In conclusion, the outcomes of this study suggest that transepithelial topography- guided surface ablation, which integrates epithelial debridement and refractive error correction into a single custom ablation, is safe, effective, and predictable in treatment of low to moderate myopic astigmatism.
